# An evaluation of the IL 508 eight-channel blood-chemistry analyser

**DOI:** 10.1155/S1463924682000042

**Published:** 1982

**Authors:** R. W. Logan, Evelyn M. Aitchison, Elizabeth C. Jamieson, B. Stewart, F. G. Sim

**Affiliations:** Department of Biochemistry Royal Hospital for Sick Children and Queen Mother's Hospital Glasgow G3 8SJ UK


Journal of Automatic Chemistry, Volume 4, Number 1 (January–March 1982), pages 7–10

### An evaluation of the IL 508 eight-channel blood-chemistry analyser

R. W. Logan, Evelyn M. Aitchison, Elizabeth C. Jamieson, B. Stewart and F. G. Sim

*Department of Biochemistry, Royal Hospital for Sick Children and Queen Mother's Hospital, Glasgow G3 8SJ, UK*

### Description

The new IL 508 is an eight-channel discrete, selective analyser. The eight-channel configuration on the system comprises the electrolytes sodium, potassium, chloride and total carbon dioxide and the chemistry channels for measuring urea, creatinine, glucose and total protein. The instrument is modular in design with the four electrolyte channels housed on one side of the central visual display unit (VDU) and sampler unit and the four chemistry modules on the other side. The dimensions of the instrument are: height 1·25 m, width 2 m, depth 0·8 m. The instrument weighs 341 kg.

The system is intended to be used to analyse serum, heparinized plasma, urine or cerebrospinal fluid at a rate of 100 samples per hour. The actual rate of analysis is 112 samples/h if the standards used for calibration are taken into account. The central area of the instrument comprises the VDU, keyboard, thermal printer, cassette recorder and the sample platform. This platform holds six of the 10 sample racks, each of which has a capacity for 10 cups. These sample racks are magnetically coded for machine recognition. The VDU is an 8 in. diagonal screen which displays commands, results of samples and information related to a fault-finding program. The keyboard consists of four commands: ENTER, START, CLEAR and HALT, the 10 digits 0 to 9, a decimal-point key and an erase key. The keyboard is pressure-sensitive.

As this is not an alphanumeric keyboard there is no facility for the input of any patient identification apart from the laboratory accession number.

### Analytical methods

The following list gives a brief indication of each method:

| | |
|---|---|
| Sodium: | Instrumentation Laboratories' sodium electrode [1]. Indirect ion-selective electrode methodology. |
| Potassium: | Instrumentation Laboratories' potassium electrode [1]. Indirect ion-selective electrode methodology. |
| Chloride: | Mercuric thiocyanate/ferric nitrate [2]. |
| Total carbon dioxide: | Automated procedure based on van Slyke's manometric method [3]. |
| Urea nitrogen: | Urease/glutamate dehydrogenase reaction [4]. |
| Creatinine: | Alkaline picrate (kinetic) [5]. |
| Glucose: | Glucose oxidase (4-aminophenazone) [6]. |
| Total protein: | Modification of biuret method (kinetic) [7]. |

### Reagents

The reagents used during the period of evaluation were supplied by Instrumentation Laboratories. All reagents are delivered in 500 ml bottles which fit in the reagent trays on the instrument. Glucose, urea nitrogen and creatinine need to be reconstituted before use. Details regarding stability of all reagents are given in the manufacturer's instruction manual.

### Procedure

Evaluation procedures were based on a recommended scheme [8] and the equipment was not modified in any way during the trial period. Patient samples and quality-control material covering a wide range of concentrations were employed for method comparison.

*Sample size*

The total sample requirement for all eight channels is 230 μl. This is a convenient volume for the 300 μl cups commonly in use in paediatric biochemistry laboratories. Smaller volumes can, however, be used for selective analyses.

*Total carry-over*

Sample interaction was measured by analysis of 10 alternating groups containing three specimens of elevated concentration and three of low concentration, i.e. specimens A1, A2 and A3 contained a high level and were followed by specimens B1, B2 and B3 containing a low level of concentration for all channels. The results showing average A1, A3, B1 and B3 values and percentage interaction are illustrated in table 1.

*Table 1. IL 508: total carry-over.*

| Channel | Units | A1 | A3 | B1 | B3 | % interaction |
|---|---|---|---|---|---|---|
| Sodium | mmol/l | 152 | 152 | 102 | 101 | +1·96 |
| Potassium | mmol/l | 7·9 | 8·0 | 2·0 | 1·9 | +1·64 |
| Chloride | mmol/l | 127 | 128 | 69 | 67 | +3·28 |
| Total carbon dioxide | mmol/l | 51 | 52 | 14 | 12 | +5·00 |
| Urea | mmol/l | 36·3 | 36·3 | 6·5 | 6·5 | 0 |
| Creatinine | μmol/l | 929 | 927 | 97 | 99 | −0·24 |
| Glucose | mmol/l | 48·3 | 49·1 | 5·3 | 5·2 | +0·23 |
| Total protein | g/l | 64 | 64 | 41 | 41 | 0 |

*Precision*

Within-batch precision was measured at high, low and mid range levels of concentration by analysis of 40 replicates. Samples which were stored deep-frozen were employed for assessment of between-batch precision. These samples were run with each batch of patient samples on 20 separate occasions. Table 2 lists the within-batch results and table 3 the between-batch figures.

*Linearity*

Table 4 lists the ranges tested for each channel; in all cases these were found to be linear. The manufacturer's operating ranges are also quoted in table 4. Outwith these ranges a range error 'R' is displayed alongside the result. Total carbon dioxide was tested to only 40 mmol/l as this is a reasonable upper clinical level.

0142–0453/82/0401 0007 $05·00 © 1982 Taylor & Francis Ltd

*Table 2. IL 508: within-batch precision at three concentrations.*

| Channel | | Low | Medium | High |
|---|---|---|---|---|
| Sodium | Mean | 102 | 141 | 153 |
| | S.D. | 1·09 | 0·77 | 1·26 |
| | CV% | 1·07 | 0·55 | 0·82 |
| Potassium | Mean | 2·1 | 3·7 | 7·3 |
| | S.D. | 0·04 | 0·05 | 0·08 |
| | CV% | 1·92 | 1·37 | 1·09 |
| Chloride | Mean | 79 | 106 | 120 |
| | S.D. | 0·99 | 0·80 | 1·29 |
| | CV% | 1·25 | 0·75 | 1·10 |
| Carbon dioxide | Mean | 14·1 | 27·3 | 41·0 |
| | S.D. | 1·24 | 0·89 | 1·30 |
| | CV% | 8·79 | 3·26 | 3·17 |
| Urea | Mean | 2·9 | 10·4 | 36·9 |
| | S.D. | 0·11 | 0·11 | 0·27 |
| | CV% | 3·82 | 1·06 | 0·73 |
| Creatinine | Mean | 53 | 106 | 766 |
| | S.D. | 1·88 | 2·81 | 9·81 |
| | CV% | 3·51 | 2·65 | 1·28 |
| Glucose | Mean | 2·1 | 8·0 | 24·8 |
| | S.D. | 0·13 | 0·09 | 0·20 |
| | CV% | 6·05 | 1·09 | 0·81 |
| Total protein | Mean | 40·9 | 76·0 | 82·9 |
| | S.D. | 0·93 | 1·04 | 0·80 |
| | CV% | 2·27 | 1·37 | 0·97 |

*Table 3. IL 508: between-batch precision at three concentrations.*

| Channel | | Low | Medium | High |
|---|---|---|---|---|
| Sodium | Mean | 120 | 140 | 154 |
| | S.D. | 1·20 | 1·36 | 1·24 |
| | CV% | 1·00 | 0·97 | 0·81 |
| Potassium | Mean | 3·0 | 4·6 | 6·9 |
| | S.D. | 0·04 | 0·06 | 0·09 |
| | CV% | 1·33 | 1·30 | 1·30 |
| Chloride | Mean | 81 | 103 | 112 |
| | S.D. | 0·73 | 1·42 | 1·32 |
| | CV% | 0·90 | 1·38 | 1·18 |
| Total carbon dioxide | Mean | 11·9 | 20·5 | 25·0 |
| | S.D. | 1·09 | 1·20 | 1·64 |
| | CV% | 9·16 | 5·85 | 6·56 |
| Glucose | Mean | 4·1 | 11·0 | 15·7 |
| | S.D. | 0·16 | 0·30 | 0·46 |
| | CV% | 3·90 | 2·73 | 2·93 |
| Urea | Mean | 4·3 | 10·5 | 29·7 |
| | S.D. | 0·09 | 0·27 | 1·32 |
| | CV% | 2·09 | 2·57 | 4·44 |
| Creatinine | Mean | 87 | 152 | 678 |
| | S.D. | 2·39 | 5·35 | 19·80 |
| | CV% | 2·75 | 3·52 | 2·92 |
| Total protein | Mean | 35·3 | 63·3 | 72·0 |
| | S.D. | 1·07 | 1·90 | 1·43 |
| | CV% | 3·03 | 3·00 | 1·99 |

### *Accuracy of analysis*

Commercial control sera (labelled A–E) were analysed repeatedly over the period of evaluation and the average results compared with the manufacturer's target values for the appropriate methodologies (table 5). Method-comparison studies were performed by analysing 150 patient samples with the IL 508 and the instruments currently used within this hospital district. The results were subjected to regression analysis and are presented in table 6.

*Table 4. IL 508: linearity of response and manufacturer's quoted operating ranges.*

| Channel | Units | Range tested | IL 508 operating ranges |
|---|---|---|---|
| Sodium | mmol/l | 100–200 | 100–180 |
| Potassium | mmol/l | 0–10 | 1·0–9·0 |
| Chloride | mmol/l | 70–140 | 70–140 |
| Total carbon dioxide | mmol/l | 5–40 | 0–84 |
| Urea | mmol/l | 0–40 | 0–35·7 |
| Creatinine | μmol/l | 0–1350 | 0–1328 |
| Glucose | mmol/l | 0–50 | 0·3–41·7 |
| Total protein | g/l | 10–120 | 20–110 |

*Table 5. IL 508: control comparisons.*

| Channel | | Control sera: A | B | C | D | E |
|---|---|---|---|---|---|---|
| Sodium | M.V. | 142 | 151 | 130 | 140 | 153 |
| | IL 508 | 141 | 151 | 130 | 140 | 153 |
| Potassium | M.V. | 4·7 | 5·3 | 3·9 | 4·2 | 7·3 |
| | IL 508 | 4·7 | 5·4 | 3·9 | 4·3 | 7·4 |
| Chloride | M.V. | 97 | 102 | 89 | 103 | 120 |
| | IL 508 | 101 | 105 | 91 | 107 | 122 |
| Total carbon dioxide | M.V. | 20 | 24 | 19 | | |
| | IL 508 | 19 | 24 | 18 | 12 | 10 |
| Urea | M.V. | 11·0 | 29·4 | 16·0 | 5·5 | 19·2 |
| | IL 508 | 11·2 | 29·3 | 16·1 | 6·1 | 20·0 |
| Creatinine | M.V. | 167 | 351 | 667 | 86 | 815 |
| | IL 508 | 166 | 345 | 653 | 103 | 804 |
| Glucose | M.V. | 4·8 | 11·4 | 2·1 | 4·2 | 17·3 |
| | IL 508 | 4·9 | 12·3 | 2·2 | 4·3 | 17·2 |
| Total protein | M.V. | 77 | 62·5 | 63 | 61 | 55 |
| | IL 508 | 64 | 56 | 51 | 56 | 53 |

No. of determinations = 20.
M.V. = manufacturer's target values.

*Table 6. IL 508: comparison of patient results.*

| Channel | Method IL 508 versus | Range of values | Slope | Y-intercept | Correlation coefficient |
|---|---|---|---|---|---|
| Sodium | IL 743 | 110–170 | 0·86 | 18 | 0·990 |
| Potassium | IL 743 | 2–7 | 1·01 | −0·11 | 0·992 |
| Chloride | SMA 6/60 | 70–130 | 1·00 | 0·07 | 0·982 |
| Total carbon dioxide | SMA 6/60 | 15–35 | 1·06 | 0·33 | 0·941 |
| Urea | IL 919 | 1–60 | 0·98 | −0·10 | 0·996 |
| | SMA 6/60 | 1–60 | 1·03 | −0·16 | 0·999 |
| Creatinine | IL 919 | 10–800 | 0·97 | 1·46 | 0·981 |
| | SMA 6/60 | 10–800 | 1·18 | −16·2 | 0·980 |
| Glucose | IL919 | 1–30 | 0·94 | 0·55 | 0·988 |
| Total protein | SMAC | 40–90 | 1·01 | 0·27 | 0·969 |

IL 508 $(y)$ = slope $(m)$ × laboratory base method $(x)$ + intercept$(c)$.

### Results and discussion

### *Precision*

The results obtained from within-batch reproducibility studies (table 2) indicate that the instrument is capable of good reproducibility within a given batch of analyses. Some of the coefficient of variation (CV) results at low concentration are, however, not as good as expected. The results for the total carbon dioxide channel are disappointing at all concentrations, particularly at the low level.

### *Sample interaction*

Carry-over [9] is low or undetectable on all channels of the IL 508. This is as expected in a discrete analyser.

### *Accuracy of analysis*

Results for the five quality-control specimens gave good agreement between values determined on the IL 508 and target values, the exceptions were chloride where the IL results were consistently higher, and total protein where the values were lower by varying amounts. The total protein results are readily explained in that the quality-control material used was bovine and cannot be reliably assayed by a kinetic method. This is mentioned by Instrumentation Laboratories in their instruction manual. An explanation for the higher chloride results may be that there is no specimen dialysis on the IL 508. In the analysis of patient samples (table 6) good correlation was found in all channels with the exception of total carbon dioxide, which is probably due to differences in time of analysis between methods and also differences in the methods of comparison themselves. However, initial patient comparison runs showed poor correlation in the sodium, chloride and creatinine channels. Poor correlation in the sodium and chloride resulted from evaporation and, in the case of sodium, a faulty spin cup. Attempts to overcome evaporation were initially unsuccessful and an interim solution of loading no more than two racks at any time was adopted. The use of thin polycarbonate film spread over each of the sample racks also helped in avoiding factitious results due to specimen evaporation. The sample probe is robust enough to pierce the polycarbonate immediately prior to sample aspiration. As an alternative to the somewhat cumbersome task of applying polycarbonate film, container cups with a small central hole were subsequently used in conjugation with the tray-cover supplied with the instrument. The effects of evaporation were then successfully eliminated for periods up to 45 minutes. Samples in unprotected micro-sample cups were earlier found to be subject to evaporation which produced changes in plasma sodium concentration of about 2 mmol/l after only 20 min.

Drift during routine machine use was tested by running 50 samples without any intermediate calibrations. Results showed no appreciable drift on any channel.

The creatinine results were initially up to 40% lower than the SMA 6/60 values. An improved creatinine reagent, however, overcame this problem resulting in the reported correlation (table 6). This new reagent also almost completely eliminated the interference of bilirubin.

The total protein analysis was examined with a series of albumin to globulin ratios, from 1 : 5 to 5 : 1, with no variation in the total protein result. It was observed, however, that bovine controls did not react as quickly as human serum giving results which were on average 15% low. Precision runs were carried out using turbid and clear material and it was noted that with turbid material the precision was markedly reduced on all channels.

In the course of performing the patient comparison study, haemolysed, lipaemic and icteric samples were analysed, and no significant differences in results were observed between the methods.

### *Maintenance*

The IL 508 is very easy to maintain. Daily maintenance includes cleaning the sample probes, the cuvettes and running an electrode wash—the wash is routinely requested by the machine after a systems shut-down. All the dispensers are then primed with reagent before proceeding to calibrate the machine. Each week the glucose and urea dispensers are cleaned with a cleaning agent and distilled water; the thermal printer is also cleaned weekly. All the other dispensers are cleaned once a month.

### *Instruction manual*

A comprehensive operator's manual covers all aspects of the instrument. The manual deals adequately with the installation procedure; it has a section covering operation, a section explaining the programming procedure, maintenance and trouble-shooting, and an appendix covering the chemistries. There is also a section on system diagnostics. This explains the diagnostic software which is used as an aid in the detection and isolation of faults occurring in the system. The software is capable of exercising all simple machine functions, but it cannot isolate a problem on its own. The operator must interpret diagnostic results and proceed in a step-by-step manner to isolate a failing mechanism or circuit.

### *Record of machine performance*

There were very few mechanical problems with the machine during its trial period. Most of the problems were very minor, such as syringe dispensers sticking—these were easily overcome by pressing the reset button. A problem that was not cured this way was a sticking probe, which resulted in the probe not picking up correct volumes. This in turn led to very low glucose and urea results. The problem was eventually traced using the system's diagnostics.

Initially the sodium results were erratic due to the linkage connecting the drive motor to the sodium spin cup being faulty. This resulted in an incomplete evacuation of the contents of the sodium cup. This fault was rectified by the manufacturer. The roller-covers for the chemistry and electrolyte modules have a tendency on opening and closing to come apart in sections due to badly designed guide-channels. The IL 508 would be improved if it were fitted with hinged doors for access to the machine, similar to the doors on the sampler module.

Some problems were encountered in the software—namely the clock lost time and the electrode-wash signals stopped the machine. The latter problem was rectified by fitting a new board; the slow clock proved to be a programming error.

### *Running costs*

Table 7 presents a rough estimate of running costs. The estimate is based on 250 samples per day over a working year of 260 days. Staffing requirements are difficult to estimate because the instrument was not operated on a routine basis, but from previous experience a figure of 1·5 staff members would seem to be realistic.

*Table 7. Estimated running costs for the IL 508.*

| | Cost per sample in pence |
|---|---|
| Depreciation | 6·2 |
| Reagents | 10·8 |
| Reference sera | 3·1 |
| Consumables | 3·1 |
| Staff | 13·8 |
| | £0·37 |

*Table 8. IL 508: values of calibrators.*

| Calibrator | Volume ml | Sodium mmol/l | Potassium mmol/l | Chloride mmol/l | Total carbon dioxide mmol/l | Urea mmol/l | Creatine µmol/l | Glucose mmol/l | Total protein g/l |
|---|---|---|---|---|---|---|---|---|---|
| Autocal 1 | 30 | 140 | 5·0 | 100 | 25 | | | | |
| Slope cal | 7 | 120 | 2·0 | 75 | 15 | | | | |
| Chem cal 3 | 7 | | | | | 17·9 | 353 | 11·1 | |
| Total protein | 7 | | | | | | | | 50 |

### General

Operation and maintenance of the system are simple and easily learnt by staff with experience of other laboratory equipment. The machine makes fairly economical use of reagents. The saline and buffer diluents have to be replaced twice daily, thus it would be beneficial to increase the size of the reagent bottles from 500 ml to 1 l. All reagents are supplied in 500 ml bottles with the exception of glucose and urea which are two component reagents, dry powder and 250 ml diluent. These reagents have a three-day life span but are normally used within this period. It was found necessary to let the reconstituted reagents stand for 30 min. before use. Aqueous calibrators are supplied in four separate packages (see table 8). For sodium, potassium, chloride and total carbon dioxide an initial two-point calibration is undertaken followed by subsequent two-point calibrations every 60 min. (timing can be altered). Single-point calibration (Autocal 1) is performed every 12 min. For urea, creatinine, glucose and total protein single-point calibration is used, it is repeated every 24 min.

During the evaluation it was found that the total protein calibrator was unsuitable, answers were, on average, 5 g/l low for human material. Monitrol has been substituted and gives satisfactory results. Monitrol is replaced fresh daily, the other calibrators are replaced every third day. The calibrators are protected from evaporation during use by small self-sealing cellophane diaphraghms.

During our experience with the instrument no blockage ever occurred. An efficient system of laundering the probes inside and out using flush and vacuum is applied between samples. However, short sampling may occur without being brought to the operator's notice. Small samples may be analysed for as many parameters as volume allows using the selective program. The 'run list review' program may be used to edit specimen order or selected analyses during the run. If required, the run may be interrupted by the use of a halt button.

A separate sliding rack designed to hold one sample is provided which will magnetically activate a stat sample, the analysis of which is made as soon as the current analysis is complete. The machine will then return to its programmed run. The sample racks are also magnetically coded and may be loaded in any order. Individual cups can only be identified by acquisition number together with rack number.

A series of flags to indicate drift, noise, out-of-range, out-of-calibration and/or out-of-control-range can be printed against any particular result. A further series of warnings is displayed on the VDU, for examples: 'buffer diluent low', 'strip printer paper out'. On current software, 'strip printer paper out' is only a warning, the machine will continue to analyse without recording results. With the basic instrument as supplied, these results cannot be recovered. If, however, a peripheral interface board is fitted, a cassette drive may be used to store such results for subsequent printing. Most error messages will result in a machine halt. After an unacceptable calibration, one sample will already have been analysed before the halt and will display a 'C' for out-of-calibration against the appropriate channel. As initially supplied, the only permanent record of results was via the thermal printer. However, an interface board has recently been delivered and this allows direct access to an external printer and/or computer. It has been a fairly simple exercise to program a Wang 2200 to capture the data and print the results horizontally in the order required for reporting to the wards. The integral cassette-recorder unit has only just been supplied by the manufacturer.

### Conclusions

The IL 508 can be easily incorporated into a routine analytical laboratory. The instrument is capable of handling a medium to large work-load whilst retaining the ability to analyse emergency samples quickly, either during a run or from standby.

The machine has been well designed giving access to all major components via roll-up covers.

Linkage via the interface board to a laboratory computer should render the instrument a very useful and reliable laboratory tool.

### References

1. Bailey, P. L., *Analysis with Ion-Selective Electrodes* (Heyden and Son, Inc., New York, 1976).
2. Zall, D. M., Fisher, D. and Garner, M. Q., *Analytical Chemistry*, **28** (1956), 1665.
3. Tietz, N. W. (ed.), *Fundamentals of Clinical Chemistry* (W. B. Saunders, Philadelphia, 1970), p. 630.
4. Gutmann, I. and Bergmeyer, H. U., *Methods of Enzymic Analysis* (Verlag Chemie Weinheim, New York and Academic Press Inc., London, 1974), p. 1791.
5. Fabiny, D. L. and Ertingshausen, G., *Clinical Chemistry*, **17** (1971), 696.
6. Trinder, P., *Annals of Clinical Biochemistry*, **6** (1969), 24.
7. Bergkuist, C. E. and Whittemore, P., *Clinical Chemistry*, **26** (1980 A), 1057.
8. Broughton, P. M. G., Buttolph, M. A., Gowenlock, A. H., Neil, D. W. and Skeltenbury, R. G., *Journal of Clinical Pathology*, **22** (1969), 278.
9. Broughton, P. M. G., Gowenlock, A. H., McCormack, J. J. and Neil, D. W., *Annals of Clinical Biochemistry*, **11** (1974), 207.

